# An elderly woman with 'Red Man Syndrome' in association with oral vancomycin therapy: a case report

**DOI:** 10.1186/1757-1626-1-111

**Published:** 2008-08-18

**Authors:** Phillippa Bailey, Henry Gray

**Affiliations:** 1 Frenchay Hospital, Frenchay Park Road, Bristol, BS16 1LE, UK; 2 Southmead Hospital, Westbury-on-Trym, Bristol, BS10 5NB, UK

## Abstract

**Introduction:**

'Red Man Syndrome' is a recognized adverse reaction to intravenous vancomycin therapy. This case concerns an elderly woman who developed a 'Red Man Syndrome' reaction whilst on oral vancomycin therapy for Clostridium difficile (C difficile) diarrhoea. Isolated case reports exist recording this reaction in association with oral vancomycin therapy in patients with inflammatory bowel conditions or impaired renal function, of which this patient had both.

**Case Presentation:**

An 82 year old Caucasian woman who developed C. difficile diarrhoea after co-amoxiclav therapy for a urinary tract infection. She was treated with oral vancomycin therapy during which she developed a widespread erythematous rash in keeping with that of 'Red Man Syndrome'. This rash resolved on stopping the oral vancomycin.

**Conclusion:**

This case is important in the light of the increasing use of oral vancomycin to treat C. difficile diarrhoea, a rising problem in the UK. It also calls us to review our understanding of the mechanism of the 'Red Man Syndrome' reaction. It is possible that significant absorption of orally administered vancomycin occurs in the presence of an inflammatory bowel condition.

## Background

### Vancomycin and Red Man Syndrome

Vancomycin can cause two types of hypersensitivity reaction – the red man syndrome and anaphylaxis. 'Red man Syndrome' is thought to be an infusion-related reaction consisting of pruritus, an erythematous rash involving the face, neck and upper torso. Patients commonly complain of the sensations of burning and itching. Agitation, dizziness, headaches, chills, fever and perioral paresthesia are also described. The incidence of red man syndrome varies between 3.7% and 47% in infected patients [1].

The 'Red Man Syndrome' seen in association with intravenous vancomycin administration is not a true allergic reaction. It appears to be due to vancomycin-induced histamine release without involvement of preformed antibodies [2]. In animal models, vancomycin provokes histamine release from rat peritoneal mast cells [3]. There is evidence of reduced side effects associated with vancomycin when antihistamines are given with therapy. In a prospective, randomized, double-blind, placebo-controlled study in 30 patients who required vancomycin chemoprophylaxis before elective arthroplasty, oral pre-treatment with either a histamine H1 receptor antagonist (diphenhydramine 1 mg/kg) or a histamine H2 receptor antagonist (cimetidine 4 mg/kg) significantly reduced the histamine-related adverse effects of rapid vancomycin infusion [4].

### Hypothesized mechanism of reaction

It is generally understood that systemic bioavailability of orally administered vancomycin is negligible [5]. In one study, after 7 doses of oral vancomycin 250 mg every 8 hours in healthy volunteers, no vancomycin concentrations were detected in serum (sensitivity limit 0.64 micrograms/ml) or urine [6].

However, the presence of an inflammatory bowel process can result in increased absorption of oral vancomycin [7]. In patients with pseudomembranous colitis and severe renal failure, several studies have revealed that orally administered vancomycin may occasionally reach therapeutic concentrations in serum and even pose a risk of systemic toxicity [8,9].

In one of these case reports an anephric girl with C. difficile pseudomembranous colitis received oral vancomycin 250 mg every 6 hours. She developed unexplained fever and encephalopathy. Sustained serum vancomycin concentrations of 34 micrograms/ml were recorded, with Cerebrospinal fluid (CSF) concentrations of 4.3 microgram/ml. Discontinuation of the drug, along with haemodialysis caused the serum concentration to fall to 24 microgram/ml with resolution of the symptoms [9].

## Case presentation

The patient, an 82 year old Caucasian woman, was admitted to hospital with confusion. She had a number of active medical problems, including atrial fibrillation, ischaemic heart disease, chronic kidney disease, hypertension and osteoarthritis. Six years prior to admission she had undergone a right hemicolectomy for Dukes B colorectal carcinoma.

During admission she was diagnosed as having a urinary tract infection (UTI). Prior to admission her general practitioner had treated the patient with a 3 day course of trimethoprim 200 mg bd. On admission to hospital, urine culture was positive for Escherichia. coli and she was prescribed a 5 day course of oral co-amoxiclav 625 mg tds. Four days following completion of this course of antibiotics, she developed diarrhoea, which was positive for Clostridium difficile toxin. Metronidazole 400 mg tds was prescribed immediately. The patient's diarrhoea worsened, she was opening her bowels 7 times daily with a Bristol Stool classification Type 7 stool. After five days of oral metronidazole therapy, oral vancomycin 250 mg qds was started.

Four days after oral vancomycin therapy was instigated, the patient developed a widespread pruritic, confluent, erythematous rash over her chest, back, neck and thighs. She also complained of a severe headache. Dermatology review was requested and the opinion was that the rash appeared to be like that seen in 'Red Man Syndrome'. It was confirmed with nursing staff that no drug errors had been made and no vancomycin had been administered intravenously in error.

Vancomycin therapy was stopped immediately and regular antihistamines were prescribed. The rash then cleared and did not return. Rechallenge with oral vancomycin was not initiated. No other drug therapy was altered during this time, and no other potential allergens could be indentified.

On admission the patient had Chronic Kidney Disease (CKD) stage 2. When she developed C. difficile diarrhoea the patient developed acute-on-chronic renal failure, with renal function deteriorating to an equivalent of CKD stage 3. Some case reports of patients developing 'Red Man Syndrome' in association with oral vancomycin therapy have involved patients with impaired renal function, suggesting that reduced excretion of any systemically absorbed vancomycin may contribute to developing the reaction. Unfortunately, despite our request, vancomycin levels were not performed by our laboratory.

The reaction seen in our patient seemed to be the same as that seen previously with intravenous vancomycin administration – the 'Red Man Syndrome'. Review of the literature reveals a number of existing case reports describing rashes during oral vancomycin therapy, including one case of measurable serum vancomycin levels. All cases we became aware of have been described in patients in the presence of colitis or impaired renal function.

## Discussion

As described, this reaction is thought to be associated with intravenous administration of the drug, but there are isolated case reports of 'Red Man Syndrome' in association with intraperitoneal and oral administration of vancomycin [8,10,11].

## Conclusion

With increasing incidence of C. difficile pseudomembranous colitis, and the increased use of oral vancomycin therapy to treat it, reporting of cases of possible 'Red Man Syndrome' is essential. From our literature review, cases have only been reported in patients with an inflammatory bowel process. It cannot necessarily be concluded that inflammatory bowel processes are required to allow the absorption of vancomycin. It is true that it is only in these cases possible 'Red Man Syndrome' has been reported but oral vancomycin has few indications for use.

It causes us to question the mechanism of this drug reaction. Does the presence of an inflammatory bowel process allow vancomycin absorption, resulting in significant serum concentrations of the drug, made more significant if there is reduced renal excretion? Alternatively, is the presence of vancomycin in the gut able to cause histamine release without systemic absorption? With increasing use of oral vancomycin, recording of possible 'Red Man Syndrome', with measurement of serum vancomycin concentrations, is essential.

## Consent

Written informed consent was obtained from the patient for publication of this case report. A copy of the written consent is available for review by the Editor-in-Chief of this journal.

## Competing interests

The authors declare that they have no competing interests.

## Authors' contributions

HG collected the details of the case and recorded the patient data including microbiology specimens and renal function. PB was the major contributor in researching and writing the manuscript. Both authors read and approved the final manuscript.
